# Keeping community health workers in Uganda motivated: key challenges, facilitators, and preferred program inputs

**DOI:** 10.9745/GHSP-D-13-00140

**Published:** 2014-01-29

**Authors:** Aurélie Brunie, Patricia Wamala-Mucheri, Conrad Otterness, Angela Akol, Mario Chen, Leonard Bufumbo, Mark Weaver

**Affiliations:** a FHI 360, Washington, DC, USA; b FHI 360, Kampala, Uganda. Now with the Clinton Health Access Initiative, Kampala, Uganda; c FHI 360, Durham, NC, USA. Now with Community Partners International, Mae Sot, Thailand; d FHI 360, Kampala, Uganda; e FHI 360, Durham, NC, USA; f FHI 360, Durham, NC, USA. Now with the University of North Carolina at Chapel Hill, Chapel Hill, NC, USA

## Abstract

**Introduction:**

In the face of global health worker shortages, community health workers (CHWs) are an important health care delivery strategy for underserved populations. In Uganda, community-based programs often use volunteer CHWs to extend services, including family planning, in rural areas. This study examined factors related to CHW motivation and level of activity in 3 family planning programs in Uganda.

**Methods:**

Data were collected between July and August 2011, and sources comprised 183 surveys with active CHWs, in-depth interviews (IDIs) with 43 active CHWs and 5 former CHWs, and service statistics records. Surveys included a discrete choice experiment (DCE) to elicit CHW preferences for selected program inputs.

**Results:**

Service statistics indicated an average of 56 visits with family planning clients per surveyed CHW over the 3-month period prior to data collection. In the survey, new skills and knowledge, perceived impact on the community, and enhanced status were the main positive aspects of the job reported by CHWs; the main challenges related to transportation. Multivariate analyses identified 2 correlates of CHWs being highly vs. less active (in terms of number of client visits): experiencing problems with supplies and not collaborating with peers. DCE results showed that provision of a package including a T-shirt, badge, and bicycle was the program input CHWs preferred, followed by a mobile phone (without airtime). IDI data reinforced and supplemented these quantitative findings. Social prestige, social responsibility, and aspirations for other opportunities were important motivators, while main challenges related to transportation and commodity stockouts. CHWs had complex motivations for wanting better compensation, including offsetting time and transportation costs, providing for their families, and feeling appreciated for their efforts.

**Conclusion:**

Volunteer CHW programs in Uganda and elsewhere need to carefully consider appropriate combinations of financial and nonfinancial inputs for optimal results.

## Abstract (precis)

In Uganda, community-based health programs using volunteers should focus on strengthening support systems to address transportation and stockout issues and on improving links with the health structure while reinforcing effort recognition, status, and acquisition of new skills.

## INTRODUCTION

Global discussions and initiatives underscore renewed interest in the role of community health workers (CHWs) in strengthening health systems and increasing availability of community-level primary health care services, including family planning.^1^^–^^6^ In 2004 in Uganda, the physician-population ratio was 1 to 12,500.^7^ Moreover, 70% of medical doctors and 40% of nurses and midwives work in urban areas, where only 12% of the population lives.^8^ Modern contraceptive prevalence is 26%.^9^ In this context, involving CHWs through task sharing provides a mechanism for expanding family planning services to underserved populations.

Although models vary globally, studies have shown that CHW programs promote the adoption of healthy behaviors and improve access to and use of a range of health services.^1^^,^^10^^–^^13^ However, years of program experience also reveal performance and retention problems. Reported attrition rates in CHW programs range between 3% and 77% and tend to be particularly high when CHWs are volunteers.^14^ Moreover, CHWs who stay on the job do not necessarily perform to their full potential.

Community health workers help to promote healthy behavior, but keeping them motivated is often a challenge.

Motivation reflects the degree of willingness to apply and maintain efforts toward program goals.^15^ As with other health cadres, individual motivation for CHWs drives performance and job continuation, all 3 of which, in turn, are affected by individual, program/health system, and contextual factors.^15^^–^^22^ Yet CHWs are qualitatively different from professional health workers in that they typically lack formal nursing or medical training, are embedded in the community, and often are volunteers.

The evidence base to inform normative guidance specific to CHW programs remains limited. The broad categories of factors underlying CHW level of activity and continuation on the job are known and include^14^^,^^20^^–^^24^:

- Social responsibility
- Self-efficacy
- Desire for achievement
- Recognition
- Workload and responsibilities
- Training
- Supportive supervision
- Equipment and supplies
- Peer support
- Personal growth and career development opportunities
- Financial and nonfinancial incentives

However, these factors are complex, and rigorous analyses of the specific ways in which they operate and of their relative importance are lacking. Moreover, many studies focus on one specific program, while managers throughout sub-Saharan Africa are increasingly challenged to harmonize a legacy of parallel CHW programs into a coherent national system. In Uganda, for example, the Ministry of Health has begun implementing a nationwide Village Health Team strategy whereby teams of volunteers provide a government-endorsed platform for all community-based programming.^25^

This study assessed factors affecting the motivation and level of activity of CHWs providing family planning services in 1 public-sector program and 2 programs supported by nongovernmental organizations (NGOs) in Uganda. Specific objectives were to examine key challenges and facilitators to CHWs being active and staying in service and to quantify the relative importance of specified program inputs from CHWs' perspectives.

## METHODS

### Design and Selection Procedures

We conducted a cross-sectional, mixed-methods study, including a structured survey and in-depth interviews (IDIs) with CHWs from 3 family planning programs covering 7 districts in Uganda. We selected family planning programs purposively to represent different cultural and programmatic realities, while taking into account program manager support for research and logistics. We included a public-sector program (active in 2 districts), an NGO-supported program (2 districts), and a program that had recently transitioned from an NGO to the public sector (3 districts). All programs offered the same contraceptive method mix, including condoms, pills, and injectables (although not all CHWs within a program provided injectables) (Box).

**BOX. Key Responsibilities of Community Health Workers**

- Promote family planning through health education talks with community members.
- Conduct health talks on family planning at health centers with mothers attending different clinics (for example, outpatient department, antenatal care, and postnatal care).
- Distribute contraceptive methods, including condoms, pills, and, for CHWs who are trained, injectables.
- Refer clients to the health center for management of side effects and other contraceptive methods.
- Mobilize communities for surgical camps that offer long-acting and permanent methods.

CHWs are linked to a nearby health center for supervision, referral management, and commodity supply. We listed all supporting health centers and obtained from program managers the estimated numbers of eligible CHWs linked with each health center. Eligibility criteria included: (1) having 1 year of experience distributing contraceptives, and (2) attending the last supervisory meeting or having a documented excuse for missing it.

The NGO-supported program had the most CHWs; the public-sector program was the smallest. In the largest program, we randomly selected health centers sequentially until the cumulative number of CHWs reached the target sample size; all CHWs reporting to those health centers were invited to participate, along with all CHWs in the other 2 smaller programs. For each program, we randomly assigned a subset of CHWs from each health center in the largest district to participate in an IDI; all others participated in a survey. Within these same districts, convenience samples of former CHWs from the public-sector and NGO-supported programs who still resided in their community of origin were also selected for an IDI.

### Sample Size and Data Collection

Based on estimates of the number of eligible CHWs, we assumed that 39 and 80 surveys, respectively, could be completed in the 2 smaller programs. We estimated that completing 76 surveys in the largest program would permit detecting a meaningful effect size (a 0.45 standard deviation difference between the 2 larger programs and a 0.55 standard deviation difference when compared with the smallest program) for the primary outcome, CHW level of activity, with 80% power and 5% significance level. Target numbers of IDIs were set to 14 per district in order to reach saturation.^26^

Data were collected in Luganda, Lusoga, and Samia in July and August 2011. Trained research assistants interviewed active CHWs at their supporting health center at a prearranged time. Health center supervisors helped contact former CHWs, who were interviewed in their homes.

As per local ethics guidelines, all participants received a small stipend to compensate them for their time (approximately US$4); the amount is consistent with the refund CHWs typically receive for attending program meetings.

IDIs were recorded, translated into English, and typed into word-processing files. We extracted service statistics from CHW records at the time of the interview. When CHWs failed to bring their records, we made a second attempt to collect service data at a subsequent supervisory meeting.

The Uganda National Council for Science and Technology and FHI 360's Protection of Human Subjects Committee approved this study.

### Quantitative Analysis Methods

The survey included questions on sociodemographic characteristics, supporting mechanisms (training, supervision, and supplies), recognition and incentives, and perspectives on CHW work. Fifty-one Likert-scale items examined CHW motivation (15 items) and factors thought to influence it (36 items).

The main outcome was CHWs' level of activity (highly active versus less active). We used service statistics to calculate the total number of visits with new or revisit family planning clients receiving any method between April and June 2011. To ensure that CHWs are classified fairly based on the setting in which they operate, we identified groupings of health centers located in sub-counties with comparable terrain and population density profiles. Within each grouping, we classified CHWs whose number of visits fell above or at the median for the CHWs associated with the facilities in the group as highly active, and others as less active. Our initial intent was to derive this measure from survey data on clients served adjusted by catchment area size. However, descriptive statistics raised concerns regarding the reliability of those survey items, and due to a redistricting process, it was also not possible to obtain catchment population data from other sources.

We conducted 2 exploratory factor analyses, one on motivational outcomes and one on motivational determinants, using principal factors extractions with oblique promax rotations to reduce Likert-scale data. Criteria for extraction included the scree test and percentage of variance (75%).^27^^,^^28^ We discarded items with little or no variation, factor loadings under 0.3, or cross-loadings. We used scoring coefficients to estimate factor scores to serve as variables in subsequent analyses. Bivariate analyses using chi-square tests examined the association between 18 variables and level of activity. Variable selection was informed by the available literature and descriptive analyses. Multivariate analyses using logistic regression included variables found significant (*P* < .10) in bivariate analyses and controlled for 7 other variables we identified as theoretically important. Bivariate and multivariate analyses adjusted for sampling weights and clustering effects at the health center level.

The survey included a Discrete Choice Experiment (DCE) to determine the relative importance CHWs place on selected program inputs that may affect motivation. DCE is a stated preference method, whereby respondents are asked to choose their preferred alternative between pairs of hypothetical scenarios (here, competing programs) characterized by several attributes (here, program inputs).^29^^–^^32^ Based on a literature review and a meeting with stakeholders working with CHWs in Uganda, we selected 5 program inputs and 2 to 3 appropriate levels for each (Table 1). We used a SAS macro (%ChoiceEff) to select 24 program profiles constructed from these attributes and levels and organized them into 12 choice pairs to produce a fractional factorial design.^33^ We evenly divided the 12 pairs into 4 groups of 3, and randomly assigned each CHW to one group for the survey. Mixed logit modeling produced a weighted ranking of program inputs. This approach models the choice probabilities with a mixture of logits and accounts for the paired data and multiple responses per CHW.^34^^,^^35^ We included all attributes as random effects, except for mobile phone, which we included as fixed effect to simplify the model and improve convergence in subgroup analyses (results not presented). This was justified by the small variance estimate of estimated coefficients for this attribute in the overall model. We adjusted DCE data analyses for sampling weights but ignored clustering by health center to simplify the models.

**TABLE 1.** Program Inputs and Levels Used in the Discrete Choice Experiment (DCE)

**Program Inputs**	**Level 1**	**Level 2**	**Level 3**
Training	5-day initial training and 3-day supervised practicum at health center	Same as Level 1 + 3-day refresher training once a year	N/A
Supervision	Monthly CHW meetings at health center	Same as Level 1 + quarterly visit by health center staff in the community	N/A
Incentives	CHW kit with gumboots, raincoat, job aids, and stationery	CHW kit + T-shirt + badge	CHW kit + T-shirt + badge + bicycle
Transportation refund	5,000 UGX for each meeting	10,000 UGX for each meeting	N/A
Communication	No mobile phone	Mobile phone without airtime	N/A

Abbreviations: CHW, community health worker; N/A, not applicable; UGX, Ugandan Shilling.

DCE data were analyzed in Stata 10; all other analyses were conducted in SAS 9.2.

### Qualitative Analysis Methods

We uploaded IDI data in NVivo 9 for analysis. We followed an iterative process of reading, coding, data display, and reduction.^36^ We developed matrices in Excel to summarize participants' responses to important thematic concepts and to examine similarities and differences between CHWs across programs and between men and women. Findings that differ across these groups are noted in the results section.

Overall data quality was high. Following initial questions guided by the study's themes, interviewers solicited richer responses through probing in a manner that was responsive to participants' answers. Biased responses arising from leading questions were noted. Upon completing quantitative and qualitative analyses, we compared results thematically.

## RESULTS

Completed interviews with active CHWs included 183 surveys and 43 IDIs (Table 2); the combined response rate was 91%. We obtained complete service statistics for 157 of the survey participants. Five former CHWs participated in an IDI.

**TABLE 2.** Program Characteristics and Number of Interviews Conducted, by Program Type

	**Active CHWs**			**Former CHWs**
**Program Type**	**No. of Surveys^a^**	**No. of IDIs**	**Total No. Interviewed/ Total No. in Program**	**No. of IDIs**
Public sector (2 districts)	35	13	48/48	3
Former NGO (3 districts)	82	14	96/112	0
NGO (2 districts)	66	16	82/203	2
Total	183	43	226/363	5

Abbreviations: CHWs, community health workers; IDIs, in-depth interviews; NGO, nongovernmental organization.

a Surveys included a Discrete Choice Experiment (DCE).

Survey respondents, on average, were 41.3 years old and had 5.5 children. Half were women, and 82.8% were currently married or cohabitating. All had attended at least primary school, with 72.6% continuing to secondary education or higher. On average, they had been providing family planning for 5.6 years; 54.4% of CHWs in the NGO program and nearly all in the other 2 programs offered injectables in addition to condoms and pills. Most (92.7%) provided other services besides family planning. Service statistics showed an average of 56 client visits per CHW between April and June 2011. The mean number of visits was the highest in the former NGO program (68), followed by the NGO (52) and public-sector (42) programs.

### Survey Results

The **positive aspects** of the work CHWs reported most frequently were:

- Acquiring new skills and knowledge (73.9%)
- Perceived impact (67.4%)
- Enhanced status (66.1%)
- Helping community members (53.7%)
- Working with health care professionals (38.2%)
- Feeling competent (20.9%)

Main positive aspects reported by CHWs included acquiring new skills, impact on the community, and enhanced status.

While there were commonalities across programs, specific factors varied in relative importance. For example, enhanced status and new skills were mentioned equally in the former NGO program (79.3%), whereas the feeling of having an impact was the most important (73.5%) in the public sector.

The **most common challenges** were transport-related:

- Transport/difficulty reaching clients (74.4%)
- Insufficient transport refund for supervisory meetings (60.1%)

Main challenges reported by CHWs related to transport.

In the former NGO program, stockouts of contraceptive commodities were also frequently mentioned (50%), while lack of compensation was important among public-sector CHWs (50%).

Most CHWs (89.2%) had received some recognition or support from their community in the past year. The main examples were being called “doctor” (73.5%), being consulted for advice on a range of health issues (70.5%), time to address family planning at community meetings (48.4%), gifts/food/labor support (35.8%), and being thanked publicly (33.7%).

Similarly, 70.9% of CHWs had received recognition or support from a supporting health facility in the past year; 21.5% of CHWs, mostly from the NGO-related programs, reported having no supporting health center. Among those linked to a facility, the main signs of appreciation were priority treatment for self or family (73.5%), priority treatment for clients referred (57.8%), acknowledgments (52.4%), being asked to provide family planning services at the health center (35.1%), selection for paid activities (26.6%), and receiving supplies for personal use (18.3%).

Most survey respondents (89.9%) had never thought of leaving the program. This was particularly high in the public sector (97.1%). Among those who had thought about leaving, dissatisfaction with compensation was the most common main reason; an obligation to serve the community was the most common main reason for staying. Factor analysis results on motivation indicate that motivation was high overall. For example, 98% of CHWs agreed with the statement, “I am satisfied with being a family planning CHW.” For motivational determinants, 10 items were arranged in 2 factors interpreted as barriers and facilitators (Table 3).

**TABLE 3.** Item Means, Grouped by Factor, by CHW Level of Activity

	**Total (N = 183)**	**Highly Active (n = 88)**	**Less Active (n = 69)**
**Motivational Outcomes**			
Overall, I am very satisfied with being an FP CHW.	3.78	3.73	3.85
I would recommend to my children that they become CHWs.	3.50	3.57	3.48
I am glad to be working as an FP CHW rather than in some other volunteer position in the community.	3.20	3.13	3.40
I feel very little commitment to the FP CHW program.^a^	1.35	1.39	1.33
I enjoy working in my community to make it better, even if it is without pay.	3.77	3.81	3.74
I do not think that it makes good sense to spend any time working in my community without payment.^a^	1.55	1.46	1.57
I have no intention to keep doing my FP CHW work without pay.^a^	1.42	1.36	1.40
**Motivational Determinants**			
**Barriers**			
My FP CHW work takes so much time that I am worried about how to support myself and my family.^a^	2.13	2.03	2.07
My family complains about the demands of my FP CHW work.^a^	1.53	1.65	1.39
Serving as an FP CHW will not help my chances of getting a better job in the future.^a^	1.45	1.51	1.47
Talking about FP with my clients is very embarrassing.^a^	1.27	1.24	1.33
It is difficult to help clients find the method that is right for them.^a^	1.54	1.37	1.59
Contraceptive use often makes people sick.^a^	2.16	2.21	2.05
**Facilitators**			
Overall, my supervisors provide helpful feedback and support.	3.77	3.72	3.74
I can probably perform most of my activities without additional training.	2.51	2.50	2.58
The FP CHW program provides all the equipment and material I need to do my job well.	3.19	3.12	3.35
It is easy to find women who are interested in receiving an FP method in this community.	3.67	3.64	3.75

Abbreviations: CHW, community health worker; FP, family planning.

Level of activity of CHWs was based on data from service statistics, which were available for 157 CHWs.

Items were scored from 1 = “Disagree a lot” to 4 = “Agree a lot.” Nonresponses varied across items. Weighted means are reported.

a Item was reverse-scored before factor analysis.

In bivariate analyses, highly active CHWs were more likely than less active CHWs to have no prior volunteer experience, to have experienced problems resupplying from the health center, and to not collaborate with other CHWs (Table 4). In the multivariate model, only problems with supplies and collaboration with peers retained significance (Table 5).

**TABLE 4.** Selected Characteristics of Survey Respondents, by Level of Activity

**Characteristics**	**Total**	**Highly Active**	**Less Active**	***P* Value**
	**(N = 183)**	**(n = 88)**	**(n = 69)**	
Age, y, mean (SE)	41.3 (0.8)	41.1 (1.1)	40.9 (1.2)	.89
No. of living children, mean (SE)	5.5 (0.2)	5.2 (0.2)	6.0 (0.5)	.23
Education, %				
Primary	27.4	26.1	37.4	.17
Secondary or higher	72.6	73.9	62.6	
Sex, %				
Female	49.8	59.1	48.8	.47
Male	50.2	40.9	51.2	
No. of years as an FP CHW, mean (SE)	5.6 (0.4)	5.5 (0.4)	5.3 (0.4)	.83
Provides other services besides FP, %				
No	7.3	4.8	9.4	.31
Yes	92.7	95.2	90.6	
Prior volunteer experience, %				
No	10.9	12.2	5.3	.05
Yes	89.1	87.8	94.7	
Received refresher training in past year, %				
No	26.1	27.2	32.9	.50
Yes	73.9	72.8	67.1	
Ever received supervision from HC staff, %				
Never	34.0	39.4	36.7	.82
Ever	66.0	60.9	63.3	
Received supervisory visits in community in the past year, %				
No	41.3	36.0	50.8	.23
Yes	58.7	64.0	49.2	
Problems with supplies, %				
No	36.1	26.7	41.2	.01
Yes	63.9	73.3	58.8	
Received recognition/support from community in the past year, %				
No	10.9	10.0	12.0	.75
Yes	89.1	90.0	88.0	
Received incentive from NGO or district in the past year, %				
No	46.2	45.2	47.4	.81
Yes	53.8	54.8	52.3	
Collaboration with other CHWs, %				
No	37.1	46.8	24.7	.03
Yes	62.9	53.2	75.3	
Time to HC, h, mean (SE)	1.2 (0.1)	1.3 (0.1)	1.3 (0.1)	.88
Motivation, mean (SE)	-0.08 (0.1)	-0.05 (0.1)	-0.04 (0.1)	.95
Barriers, mean (SE)	-0.05 (0.1)	-0.04 (0.1)	-0.04 (0.1)	.96
Facilitators, mean (SE)	-0.02 (0.1)	-0.05 (0.1)	0.07 (0.1)	.41

Abbreviations: CHW, community health worker; FP, family planning; HC, health center; NGO, nongovernmental organization; SE, standard error.

Level of activity of CHWs was based on data from service statistics, which were available for 157 CHWs.

Nonresponses varied across items. Weighted percentages and means are reported.

**TABLE 5.** Factors Associated With CHW Level of Activity in Logistic Regression Analysis (N = 156)

**Characteristics^a^**	**Odds Ratio (95% CI)**
Demographic	
Male^b^	0.63 (0.23–1.76)
Secondary or higher education^b^	2.30 (0.75–7.01)
Age, y	1.04 (0.99–1.08)
Situational	
Prior volunteer experience^b^	0.67 (0.26–1.72)
Travel time to health center, h	0.98 (0.68–1.43)
Work	
Problems with supplies^b^	2.22 (1.32–3.75)
Collaboration with other CHWs^b^	0.33 (0.13–0.86)
Motivational outcomes and determinants	
Motivation	1.25 (0.70–2.23)
Barriers	1.07 (0.67–1.72)
Facilitators	0.67 (0.40–1.14)

Abbreviations: CHW, community health worker; CI, confidence interval.

a Control variables were education, sex, age, time to health center, motivation, barriers, and facilitators.

b Indicator variable. For male/sex, the reference is female. For education, the reference is primary education. Other variables are yes/no binary variables, with “no” as the reference level.

Table 6 presents estimated means and standard deviations of the mixed logit model coefficients for program inputs included in the DCE. Mean coefficient estimates indicate the relative importance of program inputs; ranks were based on significance first and mean coefficient magnitude second. Four inputs were statistically significant factors influencing the choice of program. Provision of a package with T-shirt, badge, and bicycle had the largest influence on CHWs' choice, on average. A mobile phone (without airtime) ranked second. The ratio of the mean coefficients permits comparing inputs directly; overall CHWs preferred the first incentive package 4 times more than the mobile phone. An increased transport refund and the addition of a yearly refresher training were also significant in persuading CHWs to select a program. When compared with the mean estimates, the standard deviations suggest fairly homogeneous preferences for the T-shirt, badge, and bicycle package, but more heterogeneity for other attributes.

CHWs preferred a package with a T-shirt, badge, and bicycle, followed by a mobile phone.

**TABLE 6.** Mixed Logit Model Results for Program Inputs Influencing CHW Preferences in the Discrete Choice Experiment (N = 182)

**Program Input**	**Model Coefficients**	
	**Mean Estimate (SE)**	**Standard Deviation Estimate (SE)**
T-shirt, badge, and bicycle	3.90^a^ (1.41)	1.24 (1.45)
Mobile phone, no airtime	0.99^b^ (0.41)	…
10,000 UGX transport refund	0.77^b^ (0.35)	1.06 (0.77)
Yearly refresher training	0.73^b^ (0.34)	1.22 (0.63)
T-shirt and badge	1.97 (1.02)	1.51 (0.86)
Quarterly supervisory visits in community	0.70 (0.38)	1.15 (0.70)

Abbreviations: CHW, community health worker; SE, standard error; UGX, Ugandan Shilling.

Number of observations = 1,092.

a *P* < .01.

b *P* < .05.

### In-Depth Interviews With Active CHWs

#### Challenges

**Transport** was a major factor influencing CHW activities and their motivation. Nearly three-quarters of IDI participants said they experienced transport challenges, including trips to the health center and movements within the community to visit clients. Such challenges appeared related to livelihood concerns. Visiting clients or going to the health center for supervisory meetings or supplies took CHWs away from their other domestic or work responsibilities. Hiring a *boda boda* (bicycle taxi) to the health center reduced fatigue and travel time but had financial implications. Echoing the experiences of many others, a 49-year-old woman said:

*Our main challenge is transportation. Sometimes it might be far, you might have to walk. In case you have got some money, you might hire a* boda-boda *when going or coming back so that you can reach faster.*

Although CHWs receive some **money** when they attend supervisory meetings (typically between 5,000–10,000 shillings, or about US$2–4), many perceived the amount to be insufficient. Some CHWs interpreted the purpose of this transport refund as mere compensation for travel expenditures while others had expectations of being able to purchase small items for home use.

Many CHWs thought the transportation refund they received was insufficient.

CHWs understood that their position was voluntary, but two-thirds felt they deserved some payment through an increased transport refund, or even a regular salary. These feelings were particularly prevalent in NGO-related programs. CHWs raised issues related to the opportunity cost of volunteer work and to buying necessities for their families. Some said money was important to ensure continued family support or to keep up with increased costs of living. For example, a 65-year-old man with 7 children said:

I just wish the people concerned help us with some monthly compensation because we at times quarrel with our women when you get home at the end of the day without anything, day after day, week after week, and year after year. … If there can be something small in monetary terms to help us take care of our families, even if it is not so much [that is] provided, it can help in buying some essential good at home.

At the same time, a number of CHWs suggested that payment would make them feel appreciated and boost their morale. For a few CHWs, all from NGO-related programs, this was linked to feelings of deservingness in light of their efforts, and to equity in relation to health workers. One-fifth of IDI participants said that lack of salary or insufficient transport refund had caused them to think about dropping out, particularly when they had to encroach on their personal resources.

Over half of CHWs described **stockouts** as another critical and demoralizing issue, particularly in the NGO program. Lack of supplies affected CHWs' ability to conduct their work and the relationship with clients; it also magnified transport problems when CHWs made trips to the health center in vain. For instance, a 44-year-old man said:

Every month, when we come here [at the health center], we usually go back with them [supplies] although sometimes we don't find them and it requires that you come back to the health center another day. … Transport is hard because from my home to here, I use 8,000 shillings and you may find that sometimes, I don't have it.

#### Facilitators

The relationship with the community was a key factor in keeping CHWs motivated. Nearly all IDI participants reported an **enhanced status** not only among clients but also in the larger community. Many described being called *musawo* (health professionals)—a term from which they derived pride. Other related motivators included greater access to help or information and being consulted on a range of problems.

**Commitment to serving the community** emerged as a clear theme, particularly among women. Over one-third of CHWs described some initial tensions, primarily with men, that typically eased over time—although some reported occasional ongoing resistance to family planning.

Close to half of women and one-third of men expressed **satisfaction with helping others.** While this often counterbalanced frustration over voluntarism or other challenges, data suggest that loyalty sometimes bordered on pressure. For instance, a 30-year-old man who had thought of dropping out said:

Personally, I have got so many clients so leaving the program became so hard for me. Sometimes, I would think about that, but when you are still thinking about quitting, the client would call you, “Musawo, this and that …,” and therefore you would feel so bad to quit since people needed your services.

CHWs were split between those primarily driven by a desire to serve the community and those with particular **interest in family planning.** Although a number of CHWs indicated they did not know that volunteer work would be about family planning when they agreed to being trained, over half said that this new knowledge had contributed to improving their personal lives or those of their family.

Acknowledged **aspirations for other opportunities** also contributed to keeping CHWs in service and provided an incentive in order to increase visibility. The majority of IDI participants said they hoped their work would lead to other opportunities with NGOs or with the district. Capturing the sentiments of many others, a 45-year-old woman said:

*I am hopeful that if I perform well like the way my* basawo *[health center midwives] trained me … I am very hopeful that I will advance. I have a feeling that my opportunities are still increasing. … I am known and I have acquired more knowledge.*

Aspirations for other work motivated many CHWs to stay in service.

#### Relationship With Other Health Staff and NGOs

CHWs interfaced with health center supervisors and with district-level and/or NGO staff. Overall, CHWs felt well-treated by health center staff. A number of CHWs said they felt part of a team. Several CHWs sometimes provided family planning services at the health center and enjoyed this, because it made them feel trusted by the staff and served to build community confidence in them. Yet a number of CHWs mentioned challenges, typically related to staff's lack of availability or to feeling unappreciated. In both NGO-related programs, the initial rapport was poor, and CHWs struggled to get supplies. However, CHWs said that the situation improved after NGO staff intervened.

IDIs suggest that CHWs perceived interactions with higher-level district or NGO staff as particularly important. In fact, when asked about supervision, some CHWs in NGO-related programs exclusively referred to NGO personnel, although they acknowledged also having contacts with health center staff. In one program, the supporting NGO had recently pulled out; in another, it was preparing to leave. In both cases, several CHWs identified pull-out as a discouraging factor for continuing on the job. In addition to feeling demoralized, their main issues or concerns had to do with the effect on their ability to continue receiving a transport refund and with losing the practical support received from NGO staff. In one program, for example, CHWs explained that NGO extension workers facilitated the reporting and resupply process by acting as a bridge between them and health centers.

### In-Depth Interviews With Former CHWs

Former CHWs included 4 men and 1 woman; 3 were from the public-sector program and 2 from the NGO-supported program. The challenges and facilitators they described were generally similar to those identified by active CHWs. The rationale former CHWs provided for leaving related to 1 or more of 5 factors: (1) transport; (2) supplies and related relationship issues with health center staff; (3) pursuit of personal work; (4) family relations; and (5) health problems. For example, a 52-year-old man who reported transport as one of his main challenges was eventually discouraged because of the way the midwife treated him when he went to get supplies. Explaining that she would make him wait only to tell him that supplies had run out, he said:

*For me the this kind of treatment from someone who is supposed to be guiding us was a very big blow to my work and this really made me lose all the strength I had for serving my community people for free … Much as she was the supervisor, she should have showed some respect to us in order to earn ours, but she kept treating us like we never mattered in any way. If the medicine was not there, then it would cost her nothing to tell you that instead of making you wait for long hoping to get the medicine and yet she knows it is not there*.

## DISCUSSION

### Transportation and Stockouts Hinder CHW Motivation

This study identified several factors affecting CHW motivation and level of activity. Transportation and stockouts were the main problems CHWs faced in performing their responsibilities, highlighting weaknesses in infrastructure and logistics support. By bringing services closer to communities, CHW programs eliminate transportation barriers for community members that limit their access to care. However, transportation problems are essentially shifted onto CHWs who have to travel long distances for work. In our study, challenges ensued due to a lack of adequate resources for visiting clients or reaching health centers for supervision or supplies.

Transportation problems were essentially shifted from community members to the workers who had to travel long distances to people's homes and to health centers.

Inconsistent commodities and issues with restocking hindered CHWs' ability to complete their tasks. Contraceptive stockouts, particularly for injectables, are a chronic issue in Uganda. Highly active CHWs were more likely to experience problems with supplies, perhaps because they needed to resupply more often. Qualitative data illustrate how, even when drugs are available, long distances and timing of shortages add to challenges in ensuring a regular supply. Although CHWs are supposed to pick up commodities when they convene at the health center for supervision, they may not always be able to get the necessary supplies because of health system shortages. Since programs provide a transport refund only for attending supervisory meetings, CHWs are not compensated for the time or expenditures associated with additional trips.

Issues with transportation and commodities in CHW programs have been reported elsewhere.^10^^,^^22^^,^^24^^,^^37^ However, qualitative findings add some depth to the understanding of their implications for CHWs by showing how both issues can compound each other. Moreover, DCE results suggest ways to decrease such challenges. In particular, findings indicate that CHWs value the provision of a bicycle over a small increase in transport refund.

### Volunteerism Has Both Benefits and Costs

Voluntarism among CHWs is a matter of much debate. Findings provide important insights regarding what CHWs themselves see as the benefits and costs of volunteering. Survey and IDI data indicate that the relationship to communities and acquired skills and knowledge contributed to positive attitudes toward volunteer work and mostly positive intentions to remain in service. CHWs felt recognized and appreciated by their community and displayed a strong commitment to their clients. Enhanced status and the receptivity of others to their advice galvanized intrinsic feelings of altruism and satisfaction with helping others. Other studies also highlight social prestige and social responsibility as enabling factors.^21^^–^^23^^,^^38^ Our findings reinforce the potential of public recognition as a strategy for magnifying the positive behavioral traits that underlie CHWs' commitment.

Public recognition of CHWs can help keep them motivated.

CHWs considered family planning volunteer work as an opportunity for personal growth. Training improved competencies, bringing about personal benefits through contraceptive use and hope for future work. In rural contexts with few job opportunities, acquisition of skills is often seen as a springboard for employment, and lack of career evolution can be demoralizing.^14^^,^^24^^,^^39^^,^^40^ In this study, aspirations for NGO or government work encouraged CHWs to be active and remain in service.

IDIs highlight how weaknesses in support mechanisms can increase the costs of volunteering and can demotivate CHWs. In particular, CHWs experienced frustration at expending personal resources to cover transportation costs. CHWs considered the refund insufficient to offset actual expenditures, let alone fulfill their desire to provide for their family, at least in some small way, through their work. Despite widespread awareness of the volunteer nature of the CHW role, aspirations for a regular salary were not uncommon. The importance of income was an underlying theme, with CHWs crystallizing their expectations on the transport refund as the only existing financial incentive. This is consistent with previous studies highlighting that despite being volunteers, CHWs may see their role as income-generating.^21^^,^^38^^,^^41^ Prior research has also shown that CHWs often feel disgruntled at the lack of material benefits.^22^^,^^38^^,^^39^ Our findings add to the evidence base emphasizing the complex processes underlying CHW motivation for wanting better compensation and other opportunities.

### Relationship Between CHWs and Health Structure Are Ambivalent

CHWs act as a bridge between communities and the health structure. NGO involvement adds a third element. Our sample spanned 3 programmatic contexts: public sector, NGO, and NGO turned public. CHW level of activity was the highest in NGO-related programs. However, this finding must be interpreted cautiously because of the potentially better quality of reporting in NGO-related programs and of contextual differences.

Quantitative and qualitative data show that the relationship with the community trumped recognition by supervisors for CHWs across programs, as was also found in Colombia.^42^ Moreover, although CHWs were eager to be identified with the health structure for enhanced status, relations with health center supervisors were ambivalent as volunteers sometimes felt unappreciated or struggled to obtain supplies. NGO involvement has potential for addressing some resource constraints in CHW support systems. However, findings highlight the risk of substituting rather than complementing support functions, leading to a greater sense of accountability to NGOs than to district health staff. For example, CHWs in NGO-related programs did not always identify with a supporting health facility or recognize interactions with health center staff as supervision. In addition, there was some indication that NGO support might affect financial expectations, perhaps because of greatest exposure to incentives. NGO pull-out was an important discouraging factor, stressing that sustainability in this approach is also problematic.

### Strengths and Limitations

The mixed-methods approach and the range of programs included in the study sample strengthen our ability to confidently pinpoint key challenges and facilitators for CHWs in Uganda. First, the use of mixed methods allows for a deeper understanding of the dynamics underlying CHWs' motivation to perform and remain in service. Second, the convergence of findings across methods provides confidence in the results. Third, some generalizability beyond the study sample is supported by the important commonalities in the thematic structure of results across programs. However, although they offer insights for the current rollout of the Village Health Team strategy, findings may not be directly applicable to the entire country. Our study spans 7 of Uganda's 112 districts. Moreover, recruitment of volunteers is done by communities and may or may not absorb CHWs trained earlier in sub-national programs.

Information gathered separately from program records (results not shown), although imprecise due to different record-keeping procedures and variable time frames across programs, indicate that retention rates exceeded 80% over a 2-year period. Because our study was mostly conducted with active CHWs, and because of the high retention rate in the sampled programs, we are limited in our ability to understand reasons for not continuing. While IDIs with active and former CHWs revealed similar challenges, further research including larger samples of former CHWs is needed to identify the tipping point that causes CHWs to go from feeling discouraged to actually leaving. Our measure of CHW level of activity is a crude, intermediate measure of performance that includes aspects of service use but does not capture quality of care or more distal outcomes in terms of contraceptive use dynamics or population health. Moreover, it is expressed as number of visits, rather than clients; does not include referrals; and gives equal weight to new family planning users, clients switching methods, and clients coming to resupply although the demands placed on CHWs in each case may be different.

## CONCLUSION

While specific measures must be tailored to the local context and programmatic structure, this study provides important insights for sub-Saharan Africa on the underlying dynamics affecting CHW performance and retention, and on the relative importance of program inputs from CHWs' perspectives. Upon presenting results to implementing partners working with CHWs in Uganda, a number of recommendations arose from this study. First, standard provision of a bicycle would alleviate transport challenges. Second, CHWs should receive an allowance for attending meetings; this should be separated from reimbursement for actual transport costs. Third, accountability to the community and to the health structure, as opposed to NGOs, needs to be reinforced. Fourth, better integration of CHW supplies into forecasting is needed. Finally, prominent display of CHWs' contributions to service statistics should be promoted as a motivational tool.
